# Hierarchical Affinity Engineering in Amine-Functionalized Silica Membranes for Enhanced CO_2_ Separation: A Combined Experimental and Theoretical Study

**DOI:** 10.3390/membranes15070201

**Published:** 2025-07-02

**Authors:** Zhenghua Guo, Qian Li, Kaidi Guo, Liang Yu

**Affiliations:** 1 Beijing Key Laboratory of Photoelectronic/Electrophotonic Conversion Materials, Key Laboratory of Cluster Science, Ministry of Education, Beijing Institute of Technology Chongqing Innovation Center, Advanced Research Institute of Multidisciplinary Science, School of Chemistry and Chemical Engineering, Beijing Institute of Technology, Beijing 100081, China; 2 Beijing Institute of Technology, Zhengzhou Academy of Intelligent Technology, Zhengzhou 450000, China; 3 Beijing Institute of Technology, Zhuhai 519088, China

**Keywords:** facilitated transport mechanism, CO_2_ separation, amine-functionalized silica membranes, hierarchical affinity gradient

## Abstract

Excessive carbon dioxide (CO_2_) emissions represent a critical challenge in mitigating global warming, necessitating advanced separation technologies for efficient carbon capture. Silica-based membranes have attracted significant attention due to their exceptional chemical, thermal, and mechanical stability under harsh operating conditions. In this study, we introduce a novel layered hybrid membrane designed based on amine-functionalized silica precursors, where a distinct affinity gradient is engineered by incorporating two types of amine-functionalized materials. The top layer was composed of high-affinity amine species to maximize CO_2_ sorption, while a sublayer with milder affinity facilitated smooth CO_2_ diffusion, thereby establishing a continuous solubility gradient across the membrane. A dual approach, combining comprehensive experimental testing and rigorous theoretical modeling, was employed to elucidate the underlying CO_2_ transport mechanisms. Our results reveal that the hierarchical structure significantly enhances the intrinsic driving force for CO_2_ permeation, leading to superior separation performance compared to conventional homogeneous facilitated transport membranes. This study not only provides critical insights into the design principles of affinity gradient membranes but also demonstrates their potential for scalable, high-performance CO_2_ separation in industrial applications.

## 1. Introduction

Atmospheric carbon dioxide (CO_2_) emissions—largely resulting from industrial activities and fossil fuel combustion—continue to drive global warming and climate change [1]. As CO_2_ concentrations steadily rise, the need for efficient, cost-effective carbon capture technologies becomes ever more urgent. Traditional approaches such as chemical absorption and solid sorbents have been extensively studied; however, these methods often involve high energy consumption, complex regeneration procedures, and additional operational challenges [2,3,4]. In recent years, membrane-based separation has emerged as a promising alternative due to its lower energy demands, operational simplicity, and compact footprint [3,4,5].

Among the various membrane materials, silica-based membranes have drawn significant attention because of their excellent chemical, thermal, and mechanical stability under harsh operating conditions. Typically fabricated via sol–gel processes, these membranes possess well-defined microporous structures with pore sizes below 7 Å, enabling effective molecular sieving even at elevated temperatures and pressures [6,7]. Nonetheless, the close kinetic diameters of CO_2_ (3.3 Å) and nitrogen (N_2_, 3.64 Å) make it difficult for silica membranes relying solely on size exclusion to achieve high selectivity [6,8,9]. To address this limitation, recent research has focused on chemical modifications that enhance the intrinsic affinity of silica membranes for CO_2_. In particular, the incorporation of amine groups into the silica network has proven effective. Amines can reversibly interact with CO_2_, forming carbamate or bicarbonate species, thereby increasing CO_2_ solubility and promoting a facilitated transport mechanism [7,10,11,12]. Various methods such as impregnation, co-condensation, and post-synthesis grafting have been used to introduce amine functionalities [7,12,13]. Although these modifications generally improve CO_2_ adsorption, they typically yield homogeneous membranes in which the amine groups are uniformly distributed. While a uniform distribution can boost CO_2_ capture at the membrane surface, excessively strong interactions may slow CO_2_ desorption and hinder overall flux across the membrane [6,7,12].

A critical parameter influencing the performance of amine-functionalized membranes is the type of amine used. Primary and secondary amines tend to interact strongly with CO_2_, forming stable carbamate bonds that enhance selectivity but may impede rapid diffusion. Conversely, tertiary amines and sterically hindered amines exhibit weaker interaction with CO_2_. This results in faster adsorption–desorption kinetics but often at the expense of lower CO_2_ sorption capacity [14]. The inherent trade-off between high CO_2_ affinity and fast diffusion has therefore posed a significant challenge in designing membranes that deliver both high permeance and selectivity.

**Scheme 1:**
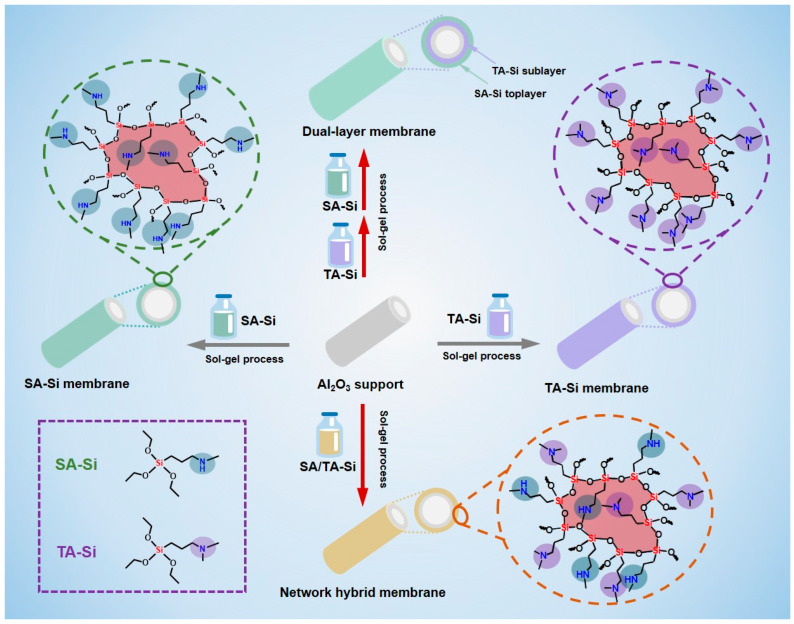
Illustration of the fabrication process of single-component amine-functionalized organosilicon membranes (SA-Si and TA-Si), network hybrid membranes (SA/TA-Si), and dual-layer membranes.

In light of these challenges, we propose a novel hierarchical affinity engineering approach to design amine-functionalized silica membranes that can better balance CO_2_ adsorption and diffusion (Scheme 1). In our design, the membrane is constructed as a layered hybrid structure where two distinct types of amine-functionalized materials are spatially distributed. The top layer contains high-affinity amine groups designed to capture CO_2_ efficiently at the feed interface. Beneath this layer, a sublayer with lower-affinity amine groups facilitates rapid CO_2_ diffusion by reducing binding energy and lowering transport resistance. This gradient creates a continuous solubility profile across the membrane, which in turn enhances the driving force for CO_2_ permeation without sacrificing selectivity.

Our approach builds on previous work that has shown promising results for both homogeneous amine–silica membranes and membranes with modified pore structures [6,7,15]. By combining layers with different CO_2_ affinities, our membrane is designed to overcome the inherent trade-off between strong CO_2_ binding (which enhances selectivity) and the need for rapid diffusion (which boosts permeance). The top high-affinity layer ensures that a sufficient amount of CO_2_ is initially captured, while the sublayer with milder affinity minimizes the resistance for CO_2_ transport, leading to an overall enhancement in CO_2_ permeance. The performance of the hierarchical membrane is evaluated using a dual approach. Experimentally, membranes are fabricated via a sol–gel process under mild conditions that help retain the amine functionalities. Gas permeation tests, conducted over a range of temperatures and pressures, measure the CO_2_ permeance and CO_2_/N_2_ (or CO_2_/CH_4_) selectivity. The results are compared with those from conventional homogeneous membranes. In parallel, theoretical modeling—including the dual-mode sorption model and molecular simulations—is used to elucidate the transport mechanisms. The combined experimental–theoretical analysis provides a detailed understanding of how the affinity gradient enhances CO_2_ transport and validates the design concept. The hierarchical affinity gradient strategy not only addresses the limitations of conventional amine-functionalized silica membranes but also paves the path for the development of scalable, high-performance membranes for carbon capture and other gas separation applications.

## 2. Experimental Section

### 2.1. Preparation of Amine-Functionalized Silica Sols, Xerogel Powders, and Membranes

Amine-functionalized silica sols were synthesized by hydrolyzing and condensing amine-functionalized precursors (SA-Si, TA-Si, and their mixtures) under conditions similar to those previously reported [6]. In a typical procedure, the precursor was prehydrolyzed in an EtOH/H_2_O/HCl system, with ethanol serving as the solvent and HCl as the catalyst. The initial molar ratio of Si/H_2_O/HCl was maintained at 1:120:0.3, and the mass concentration of the amine–silica precursor was adjusted to 3 wt.% with ethanol. The prehydrolysis reaction was carried out at room temperature for 12 h to ensure sufficient stabilization of the sol, after which the sols were stored in a refrigerator at 4 °C. Xerogel powders were obtained from these sols by solvent evaporation in Petri dishes at 50 °C and were named according to their precursor.

Porous α-alumina tubes (porosity: 50%, average pore size: 1 µm, length: 100 mm, outer diameter: 10 mm), provided by the Nikkato Corporation (Osaka, Japan), were used as supports. A SiO_2_–ZrO_2_ composite colloidal sol (2 wt.%), prepared as described elsewhere [16], was used as a binder for coating α-alumina particle layers and forming a SiO_2_–ZrO_2_ sublayer. α-Alumina particles with average diameters of 2 µm and 0.2 µm were dispersed (10 wt.%) in the colloidal sol using ultrasonic treatment. These dispersions were sequentially coated onto the outer surface of the porous support, with each coating followed by calcination at 550 °C for 15 min to form two intermediate layers. This coating procedure was repeated several times to ensure adequate coverage and to minimize large pores that could lead to pinholes. Next, the SiO_2_–ZrO_2_ sol was diluted to 0.5 wt.% with deionized water and applied to the particle layers to form a sublayer that reduced the average pore size to approximately 1 nm, followed by calcination at 550 °C for 15 min. Finally, homogeneous membranes with a hybrid network structure were fabricated by coating the composite amine–silica sol (SA/TA-Si) at a concentration of 0.15 wt.% onto the SiO_2_–ZrO_2_ intermediate layer, followed by calcination at 250 °C under N_2_ for 1 h. This coating–calcination step was repeated twice to ensure a uniform and defect-free selective layer with controlled thickness. For the layered hybrid membranes (DLM), TA-Si and SA-Si sols (also at 0.15 wt.%) were sequentially coated—each applied only once—to maintain a total selective layer thickness comparable to that of the homogeneous membranes. The membrane thickness was primarily controlled by adjusting the sol concentration and coating steps. The uniformity of the selective layer and defect-free morphologies were achieved with thicknesses typically ranging from 80 to 120 nm. These values were used for qualitative structural comparison but not directly for permeance calculation.

### 2.2. Characterization of Amine-Functionalized Silica Sols, Xerogel Powders/Films, and Membranes

Dynamic light scattering (DLS) measurements were performed using a Malvern Zetasizer Nano ZS (Malvern Instruments, Ltd., Malvern, UK) at 25 °C to determine the sol sizes. Thermal stabilities and decomposition behaviors of the xerogel powders were characterized by thermogravimetric mass spectrometry (TG-MS, TGA-DTA-PIMS 410/S, Rigaku, Tokyo, Japan). The microstructure of xerogel powders fired at 300 °C under a N_2_ atmosphere was evaluated by nitrogen adsorption–desorption isotherms at −196 °C using BELMAX equipment (BEL JAPAN INC., Osaka, Japan); prior to analysis, samples were evacuated at 180 °C for 12 h to remove adsorbed water. CO_2_ adsorption–desorption isotherms at near-ambient temperatures were also recorded using the same equipment. The true density of xerogel powders was measured by gas displacement at 40 °C using helium as the displacement gas. X-ray diffraction (XRD) analysis was conducted on these films using a D2 PHASER diffractometer (Bruker, Bremen, Germany) with Cu K-α radiation (λ = 1.54 Å). The cross-sectional morphologies of the membranes were observed using a scanning electron microscope (SEM, JCM 5700, JEOL, Tokyo, Japan).

### 2.3. Membrane Performance Evaluation

Single-gas (He, 2.6 Å; H_2_, 2.89 Å; CO_2_, 3.3 Å; N_2_, 3.64 Å; CH_4_, 3.8 Å; CF_4_, 4.7 Å; SF_6_, 5.5 Å) permeation tests were conducted based on the conventional constant pressure/variable volume technique for temperatures ranging from 35 to 200 °C. The flow rate of the feed gas was controlled with a mass flow controller (HORIBASTEC, Kyoto, Japan), and the pressure was adjusted using a back-pressure regulator. The flow rates of both the retention side and the permeate side were measured using film flow meters (HORIBASTEC, Japan). In a typical measurement, the permeate stream (inner side) was maintained at atmospheric pressure, and the pressure drop across the membrane was maintained at ~1 bar. Prior to measurement, the membrane was allowed to outgas in a He flow of 50 cm^3^/min at 200 °C for at least 6 h to remove any possible water adsorbed in/on the membrane. The permeation measurement was conducted after the membrane reached a steady state with respect to the furnace temperature and the pressure difference between the shell and the inner side of the membrane. Values for gas permeance and ideal selectivity were calculated using Equations (1) and (2), respectively.

(1) $$P_{i}=\frac{F_{i}}{A\Delta p_{i}}$$

(2) $$\alpha_{ij}=\frac{P_{i}}{P_{j}}$$

Here, *P_i_* and *P_j_* [mol/(m^2^ s Pa)] are the permeance values for components *i* and *j*, respectively; *F_i_* [mol/s] is the molar flow rate of component *I*; *A* [m^2^] is the membrane effective area; ∆*p_i_* [Pa] is the partial pressure drop of component *i* between the shell and the inner side of the tubular membrane; and *α_ij_* is the ideal selectivity (permeance ratio) of component *i* over *j*.

## 3. Results and Discussion

### 3.1. Physicochemical Properties of Amine-Functionalized Silica Xerogel Powders

**Figure 1:**
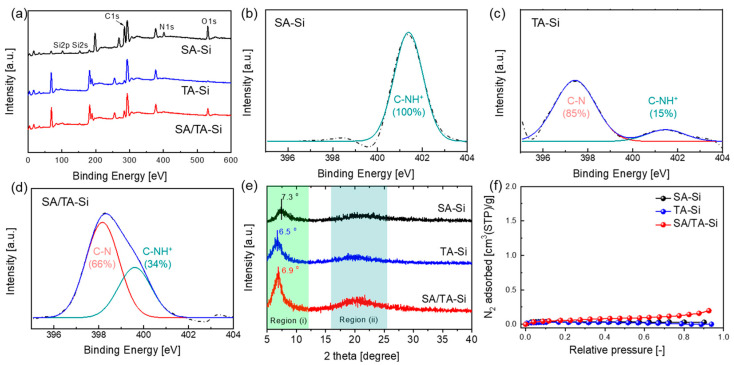
Physicochemical properties of amine-functionalized organosilicon xerogel powders: (**a**) XPS full spectra, (**b**–**d**) high-resolution XPS spectra of N 1s, (**e**) XRD patterns, and (**f**) N_2_ adsorption isotherms at 77 K.

To elucidate the physicochemical changes resulting from incorporating amine groups into the silica network, we characterized the materials using XPS, XRD, and N_2_ adsorption measurements (Figure 1). The XRD patterns of SA-Si and TA-Si xerogel powders reveal an ordered arrangement attributed to side-chain organic silane monomers forming a ladder-like structure. In the composite sample (SA/TA-Si), the 2θ peaks lie between those of SA-Si and TA-Si, indicating a uniform distribution of both components within a hybrid network. Overall, all samples exhibited a typical amorphous structure, with d-spacing in region (i) following the trend TA-Si (1.36 nm) > SA/TA-Si (1.28 nm) > SA-Si (1.21 nm). This suggests that the bulky side groups significantly influence the spacing of the ladder-like structures. XPS analysis provided insights into the elemental composition (Si, C, O, and N) and revealed similar nitrogen content among SA-Si, TA-Si, and SA/TA-Si, indicating comparable amine group densities. Detailed N1s spectra analysis showed differences in the chemical environment and basicity of the amine groups, with the protonation ratio decreasing in the following order: SA-Si > SA/TA-Si > TA-Si. The intermediate basicity of the SA/TA-Si hybrid, which combines secondary and tertiary amines, may offer tunable CO_2_ affinity in terms of adsorption capacity and adsorption heat. N_2_ adsorption measurements indicated that these xerogel powders exhibit a “nonporous” structure. This is likely due to a hybrid network formed by dead-end, flexible alkylamine chains intermingled with a rigid Si–O–Si backbone. Despite the apparent nonporosity, the dynamic free volume within the network was sufficient for gas/liquid permeation, enabling effective membrane separation. True density measurements via helium displacement showed that the densities followed the order SA-Si (1.4 ± 0.05 g/cm^3^) > TA-Si (1.20 ± 0.05 g/cm^3^) > SA/TA-Si (0.91 ± 0.01 g/cm^3^). The lower density of the SA/TA-Si composite suggests that combining both types of amine-functionalized precursors yields a more open hybrid network, which may enhance dynamic porosity and improve membrane performance.

### 3.2. CO_2_ Adsorption Properties

**Figure 2:**
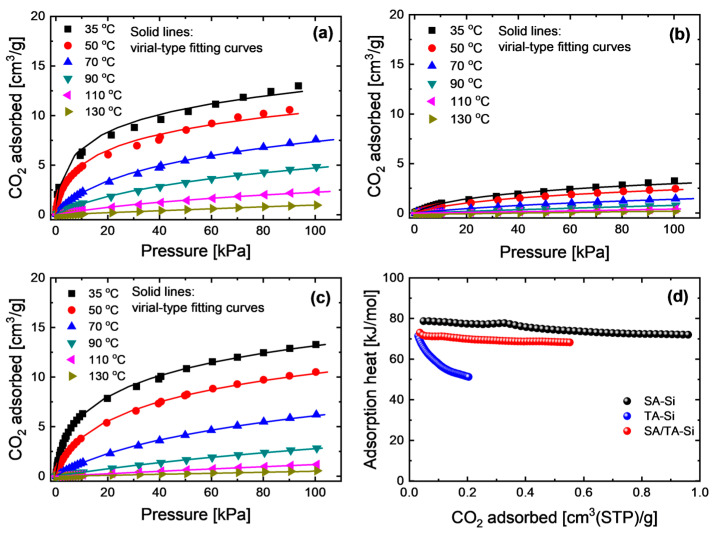
CO_2_ adsorption isotherms at a broad temperature range of (**a**) SA-Si, (**b**) TA-Si, and (**c**) SA/TA-Si, together with (**d**) their coverage-dependent isosteric adsorption heat (*Q_st_*).

CO_2_ adsorption properties were investigated over a wide temperature range (35–130 °C) to comprehensively evaluate the adsorption behavior on these xerogel powders (Figure 2). The experimentally obtained adsorption isotherms were accurately fitted using a virial-type equation, which enables the determination of reliable Henry’s constants and isosteric adsorption heats at low or zero coverage. In this model, the virial coefficients *a_i_* and *b_i_* are adjustable parameters, and for simplification, we assume *m* = *n* = 1. The coverage-dependent isosteric heat (*Q_st_*) and the zero-loading isosteric heat ($Q_{st}^{0}$) can be expressed as follows:

(3) $$ln\;p=\frac{1}{T}\sum_{i=0}^{m}a_{i}N^{i}+\sum_{i=0}^{n}b_{i}N^{i}+ln\;N$$

(4) $$Q_{\mathrm{st}}\left(N\right)=-R\left[\frac{\partial ln\;p}{\partial \left(\frac{1}{T}\right)}\right]=-R\sum_{i=0}^{m}a_{i}N^{i}$$

(5) $$Q_{st}^{0}=-R\left[\frac{\partial ln\;K}{\partial \left(\frac{1}{T}\right)}\right]=-R\;a_{0}$$

Henry’s constant (K) is directly related to the interaction of a gas–solid pair. The higher value of K indicates a stronger gas–solid interaction. Generally, Henry’s constants can be determined as the limiting slope of the adsorption isotherm and follow the Van’t Hoff expression.

(6) $$K=\underset{p\to 0}{\lim}\left(\frac{N}{p}\right)=\underset{p\to 0}{\lim}\left(\frac{dN}{dp}\right)=K_{0}\exp\left(\frac{-Q_{st}^{0}}{RT}\right)$$

(7) $$K_{0}=\exp\left(-b_{0}\right)$$

All the adsorption isotherms for the xerogel powders were well fitted by the virial-type equation across the broad temperature range, enabling the simulation of CO_2_ adsorption properties at the membrane interface over a practical operating range (typically 25–200 °C). The coverage-dependent isosteric heats exhibited the following trend: SA-Si > SA/TA-Si > TA-Si. This indicates that the affinity for CO_2_ can be tuned by mixing different amine groups.

### 3.3. Single-Gas Permeation Properties

**Figure 3:**
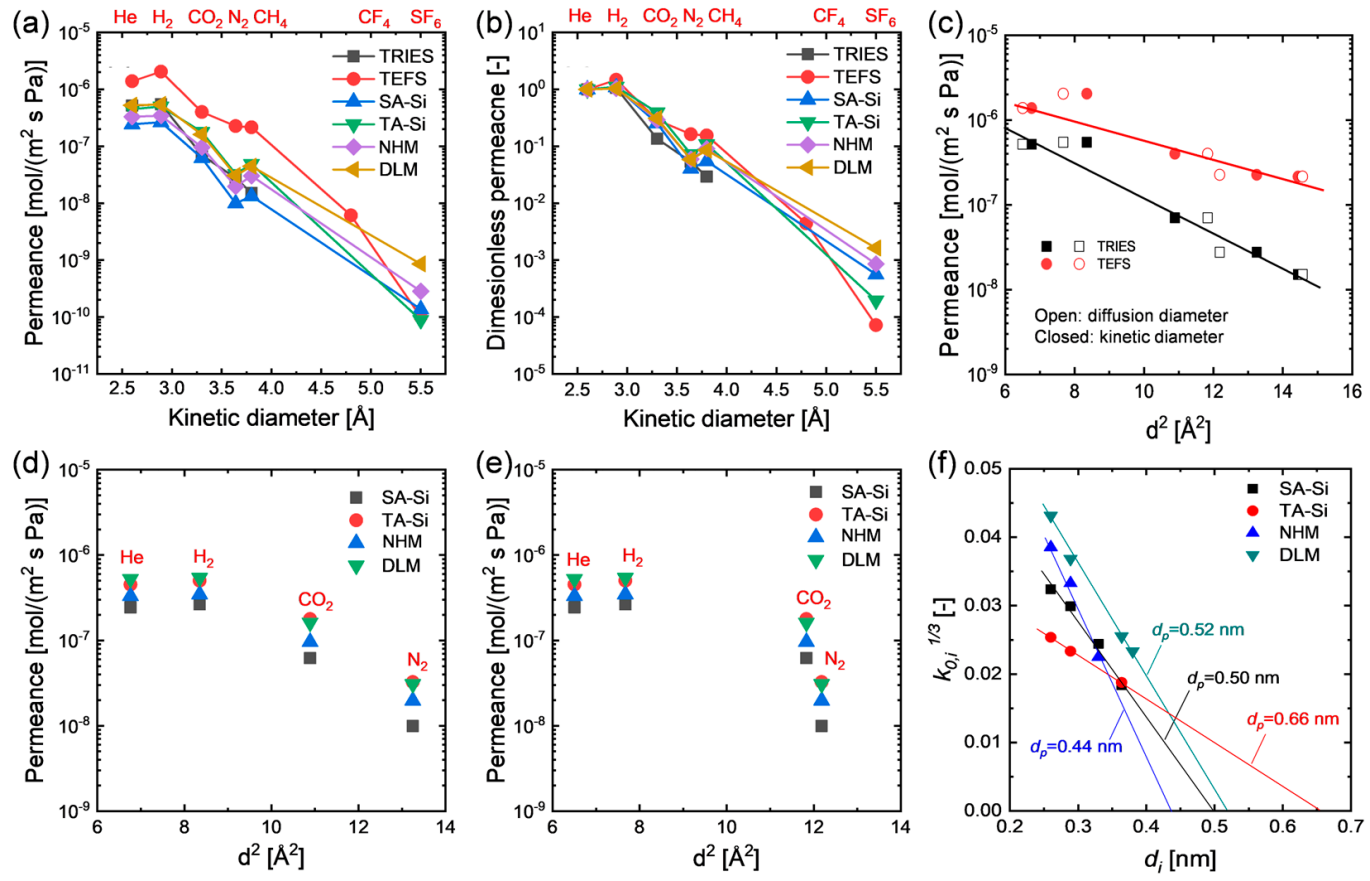
Single-gas permeation properties of single-component membranes (SA-Si and TA-Si) and hybrid membranes (NHM and DLM). Kinetic diameter dependence of (**a**) gas permeance and (**b**) dimensionless permeance at 200 °C and 200 kPa. (**c**) Gas permeance of porous organosilicon membranes derived from TRIES and TEFS membranes as a function of gas diameter squared (open: diffusion diameters; closed: kinetic diameters). Gas permeance of amine–silica membranes at 200 °C and 200 kPa as a function of (**d**) kinetic diameter squared and (**e**) diffusion diameter squared. Gas kinetic and diffusion diameters were adapted from ref. [20]. (**f**) Average pore diameter analysis based on the *K*_0_ method.

To probe the single-gas permeation properties as well as the possible microstructure of the amine–silica membranes prepared from either individual or mixed precursors in this study, gases with different diameters were adopted for single-gas permeation tests, and the results are shown in Figure 3. All the prepared membranes showed a remarkable molecular size-sieving effect even at relatively high temperatures (e.g., 200 °C), which is almost impossible for most polymeric membranes with a nonporous structure (Figure 3a,b), suggesting a thermally stable organic–inorganic hybrid structure derived from organosilicon precursors, as confirmed by their physicochemical properties. However, these membranes exhibited a comparable permeance decreasing trend with gas kinetic diameters, even compared with those membranes with porous structure prepared from organosilicon precursors consisting of pendant groups with negligible steric hindrance (e.g., ≡Si-H in TRIES and ≡Si-F in TEFS, Figure 3c). Unlike TRIES and TEFS, the precursors of SA-Si [≡Si-(CH_2_)_3_NH(CH_3_)] and TA-Si [≡Si-(CH_2_)_3_N(CH_3_)_2_] consist of pedant groups with relatively longer chains that would inevitably fill in the voids or pores produced by the rigid Si-O-Si skeleton during their condensation process. Consequently, the steric hindrance and flexibility of the pendant groups play an important role in the textural properties of the hybrid organic–inorganic network. Owing to the similar sizes of the pendant groups of SA-Si and TA-Si, they showed very similar kinetic diameter dependence of gas permeance. However, only a small difference was observed between them, with TA-Si showing a slightly loose structure due to its slightly bulky pendant groups that may accordingly enlarge the rigid network size of the Si-O-Si skeleton. Importantly, these two-novel amine–silica membranes with single-layer (NHM) and dual-layer (DLM) configurations also showed similar trends in the kinetic diameter dependence of gas permeance compared to those membranes prepared from individual precursors (Figure 3a–d). This phenomenon implies that hybrid and layered hybrid network structures cannot significantly affect the texture properties of these amine–silica membranes. Generally, the activation energy of diffusion for gases through nonporous polymeric membranes scales linearly with the gas diameter squared [17,18,19]:

(8) $$E_{d}=md_{i}^{2}+y$$

where *d_i_* is the gas diameter, and *m* and *y* are empirical parameters. Hence, at relatively higher temperatures where the solubility contribution is negligible, a linear relationship between log permeability (or permeance) and gas diameter squared can be observed. Robeson regressed diffusion diameters based on a database of experimental diffusivities and found that the gas diffusion diameters were more representative for the correlation of gas transport properties in polymers (either rubbery or glassy polymers, thermally arranged polymers, polymers of intrinsic microporosity, etc.) than prior zeolite-based kinetic diameters [20,21]. Instead, the membranes in this study with a hybrid organic–inorganic network demonstrated a more linear relationship between log permeance and gas kinetic diameter squared for the gases considered (He, H_2_, CO_2_, N_2_, and CH_4_) than the case of diffusion diameter squared. This is, nevertheless, also indicative of the strong size-sieving behavior of these membranes. However, together with the Knudsen selectivity for gas pairs of H_2_/He and CH_4_/N_2_, this phenomenon probably suggests that amine–silica membranes possess a different texture property than most popular polymeric membranes due to the rigid Si-O-Si main skeleton that plays a primary role in the diffusion of gases through these membranes. Since the pendant groups attached to the Si-O-Si network have sufficient flexibility or mobility, they cannot provide effective diffusivity selectivity for the permeate gases. The same phenomenon was also observed in the case of highly aromatic polybenzimidazoles membranes with very rigid matrix at a temperature of 190 °C [18]. In addition, the apparent average pore diameter analysis for the prepared membranes showed an order of NHM (0.44 nm) < SA-Si (0.50 nm) < DLM (0.52 nm) < TA-Si (0.66 nm), suggesting that network hybridization resulted in a denser microstructure, while layer hybridization tended to form an apparent pore size in between SA-Si (top-layer) and TA-Si (sublayer).

**Figure 4:**
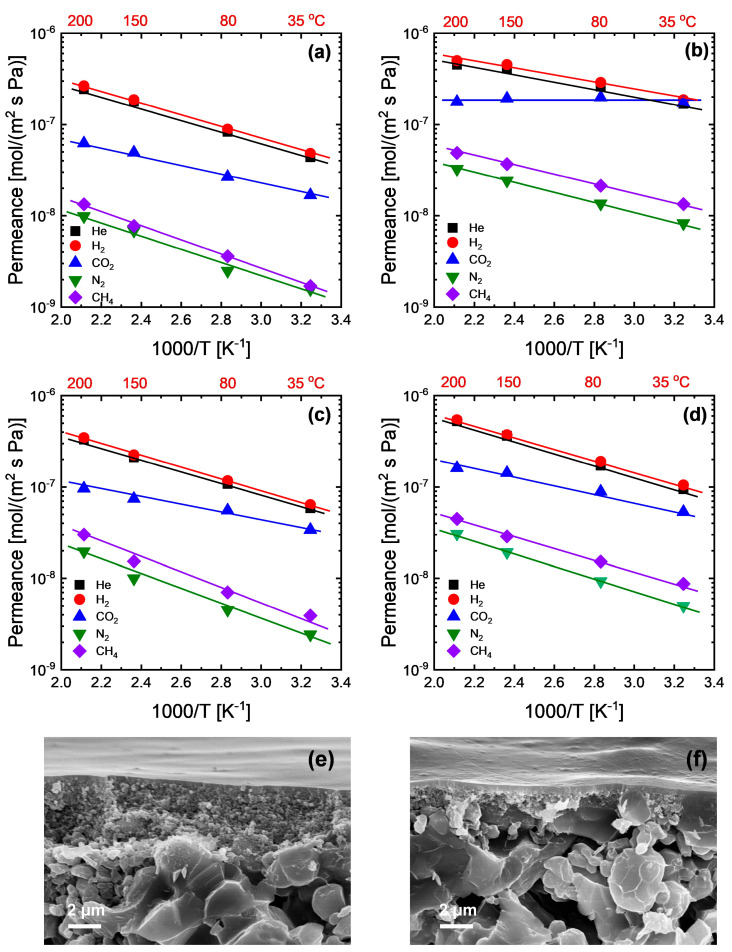
Temperature dependence of gas permeance for amine–silica membranes: (**a**) SA-Si, (**b**) TA-Si, (**c**) NHM, and (**d**) DLM. Cross-sectional SEM images of (**e**) NHM and (**f**) DLM.

Figure 4a–e show the temperature dependence of gas permeance for the membranes with hybrid (NHM) and layered hybrid (DLM) network structures; the relevant activation energies of permeation (*E_p_*) for the gases considered in this study and CO_2_/N_2_ selectivity are also tabulated in Table 1. All the analyzed gases for all the membranes showed a linear relationship between log permeance and inverse temperature that can be well described by an Arrhenius-type equation. NHM and DLM showed very similar activation energies for He and H_2_ with membrane SA-Si, indicating similar levels of diffusion barriers. It seems that the hybrid network of NHM and DLM is always dominated by the SA-Si-derived network, which is relatively denser than the TA-Si-derived network. Meanwhile, the CO_2_ permeation properties were also largely affected by the SA-Si-derived network consisting of secondary amine groups. However, on the other hand, the gas permeance always followed the order TA-Si > DLM > NHM > SA-Si. It is not so surprising that NHM showed a moderate level of permeance due to the formation of the homogenous hybrid network derived from a relatively dense network and a loose network. Figure 4e,f show a representative cross-sectional SEM image of the prepared membranes. Both NHM and DLM exhibited uniform, defect-free selective layers with a typical thickness of approximately 100 nm that were well adhered to the porous support. The comparable thicknesses of NHM and DLM, as confirmed by the SEM observation, demonstrate the successful formation of dense selective layers and provide direct visual evidence of their similar membrane morphologies. However, the DLM showed a higher permeance than the NHM at the same level of thickness. Since the permeation mechanism between homogenous single-layer membranes and dual-layer membranes may exhibit some differences due to the possible variations in pressure or concentration distribution across the membrane thickness, predictive permeation models were used for a better understanding of the mechanisms, as described in the next section.

Humidity is expected to play a significant role in the CO_2_ transport properties of amine-functionalized membranes. In the presence of water vapor, CO_2_ can react with amine groups to form bicarbonate species, thereby increasing CO_2_ uptake and facilitating its transport across the membrane. This humidified environment can enhance the overall CO_2_ separation performance, as has been widely observed for other amine-containing polymeric and hybrid membranes. Moreover, the affinity-gradient structure of our dual-layer membranes is anticipated to be especially beneficial under humid conditions: the high-affinity top layer would promote CO_2_ sorption, while the underlying low-affinity sublayer would facilitate rapid diffusion and desorption, even in the presence of water. Future work will systematically investigate the effect of water vapor on CO_2_ permeance and selectivity to fully validate these potential advantages under practical operating conditions.

### 3.4. Modeling Prediction of Hybrid Membranes

Regarding hybrid membranes, if phase separation occurs, the permeance or permeability can be well estimated based on several predictive permeation models for mixed-matrix membranes (MMMs). The dual-layer membrane is a kind of phase-separated membrane that can likely be estimated based on a series two-layer model (series model) [22]:

(9) $$P_{eff}=\frac{P_{c}P_{d}}{\phi_{c}P_{d}+\phi_{d}P_{c}}$$

where *P_eff_* is the estimated total permeability; *P_c_* and *P_d_* are the permeability of continuous and dispersed phases, respectively; and *Φ_c_* and *Φ_d_* are the volume fractions of continuous and dispersed phases, respectively. On the other hand, a parallel two-layer model (parallel) could be applied for a parallelly phase-separated membrane, which generally defines the upper limitation of a mixed-matrix membrane:

(10) $$P_{eff}=\phi_{c}P_{c}+\phi_{d}P_{d}$$

Therefore, regarding the NHM, if phase separation occurs, the series model and parallel model can define the lower and upper limitations, respectively. In addition, the geometric mean model assumes a random distribution of the phases, and the effective permeability is determined as follows [23]:

(11) $$ln\;P_{eff}=ln\;P_{c}^{\phi_{c}}+ln\;P_{d}^{\phi_{d}}$$

**Figure 5:**
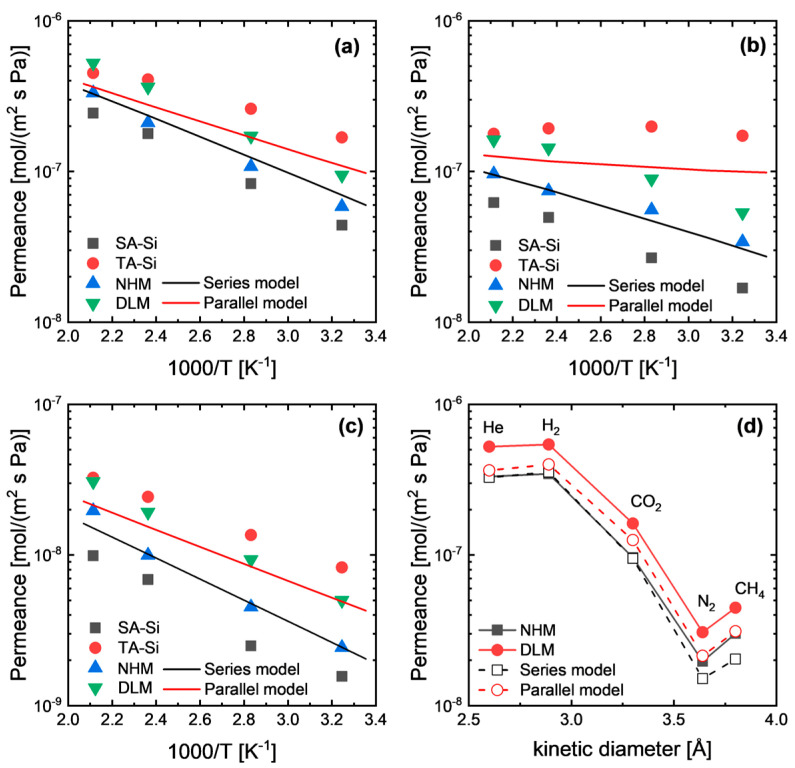
Gas permeance comparison between experimental results and modeling-based prediction for (**a**) He, (**b**) CO_2_, and (**c**) N_2_, as well as (**d**) the effect of the gas kinetic diameter.

As mentioned above, hybrid membranes (NHM and DLM) showed permeances in between SA-Si and TA-Si at a broad temperature range (Figure 5). However, NHM membranes showed comparable or even lower permeance compared with the series model’s prediction, indicating the formation of a new hybrid network that was relatively dense. In addition, surprisingly, the DLM membranes showed higher permeance than either the NHM membranes or the series model’s prediction. This is a strange phenomenon for layered membranes, since in some reported cases, the series model or resistance model can describe the gas permeation behaviors well. In this study, we hypothesized that the interfacial effect between the two layers of the DLM can partly affect the gas permeation properties due to variations in the total thickness and the possible voids formed at the interface. On the other hand, generally, the predictive permeation models for mixed-matrix membranes are mainly based on the diffusivity coefficient through each phase (continuous phase and dispersed phase), for which the solubility (or concentration) distribution across each layer is not considered. Hence, for a better understanding of gas permeation behaviors through dual-layer membranes, we sought to establish the permeation equations by considering the chemical potential distribution across the membranes.

**Figure 6:**
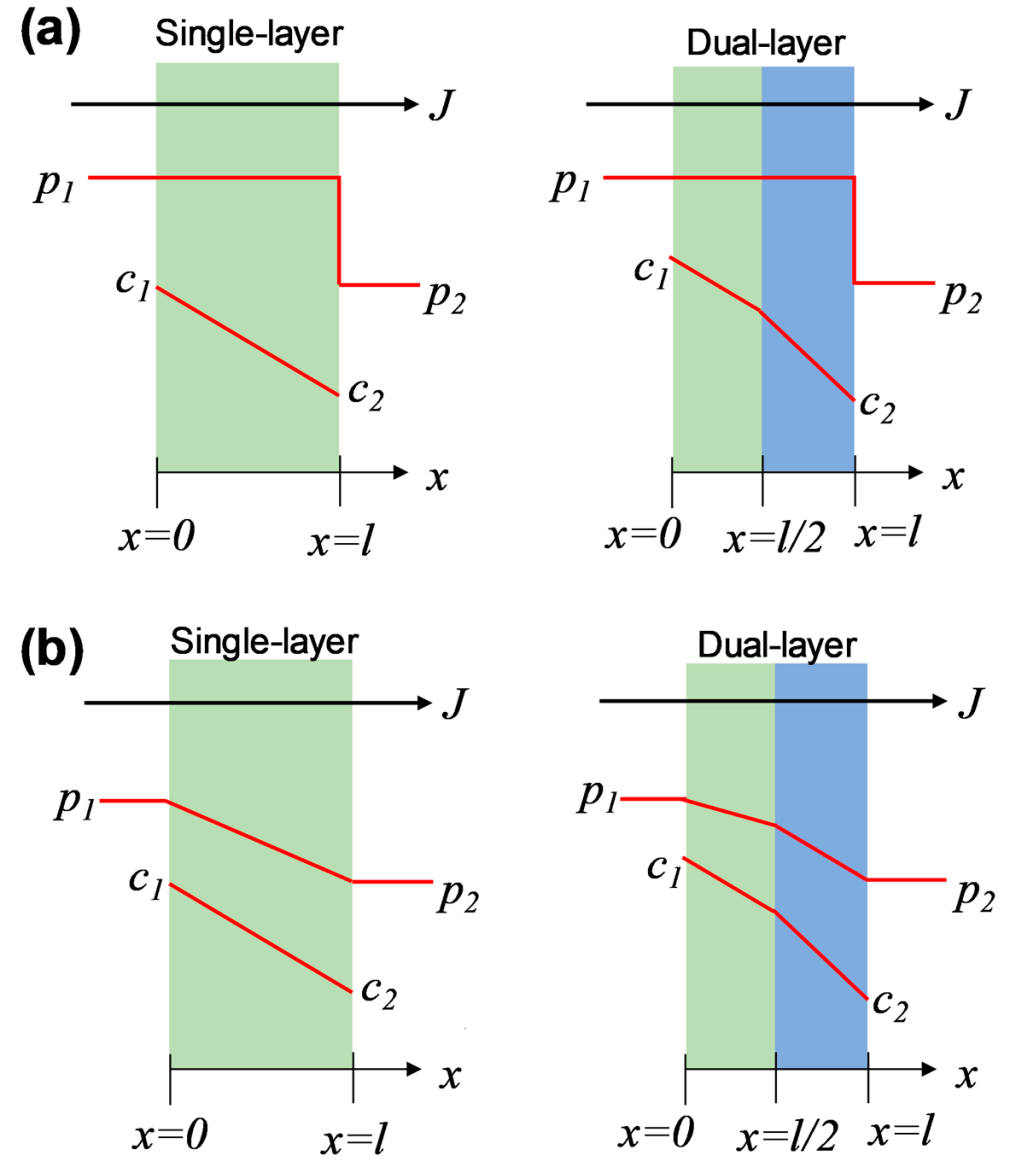
Distributions of pressure and concentration across single-layer and dual-layer membranes based on (**a**) the solution-diffusion model and (**b**) the hybrid model.

Generally, there are two typical transport models presumed to describe the chemical potential distribution in porous and nonporous membranes: the pore-flow model and the solution-diffusion model [24]. In this study, the amine–silica membranes demonstrated a hybrid network with a nonporous nature, which, however, is different from most popular dense polymeric membranes, as mentioned above. Therefore, we hypothesized that the chemical potential distribution across the dual-layer membranes follows the solution-diffusion model or a hybrid model consisting of both solution-diffusion and pore-flow models, as illustrated in Figure 6.

(12) $$J_{i}=-L_{i}\frac{d\mu_{i}}{dx}$$

where *dµ_i_*/*dx* is the gradient in chemical potential of component i, and *L_i_* is the proportionality coefficient associated with the chemical potential. Regarding compressible gases, the chemical potential can be written as follows:

(13) $$\mu_{i}=\mu_{i}+RTln\left(\gamma_{i}c_{i}\right)+RTln\frac{p}{p_{i}^{0}}$$

Since diffusion across the membrane is always significantly more difficult than adsorption–desorption at the membrane interface of both the feed and permeate sides due to the relatively dense membrane matrix or narrow pore size for gas separation membranes, we hypothesized that adsorption of the penetrated gas at the interface is always in an equilibrium state. The discussion below covers two cases: a solution-diffusion model and a hybrid model.

Case (i): solution-diffusion model

In this scenario, we assume the following conditions: (i) constant pressure across each layer and pressure drop occurs at the interface of the permeate side; (2) concentration decreases gradually across each layer; (3) the diffusion coefficients across each layer are comparable.

Then,

(14) $$\mu_{i}=\mu_{i}^{0}+RTln\left(\gamma_{i}c_{i}\right)$$

(15) $$J_{i}=-L_{i}\frac{d\mu_{i}}{dx}=-\frac{L_{i}RT}{c_{i}}\frac{dc_{i}}{dx}$$

where *γ_i_* = 1 for simplification. This equation has the same form as Fick’s first law when we replace *L_i_RT*/*c_i_* with the concentration-dependent diffusion coefficient, *D_i_*.

Thus,

(16) $$J_{i}=-D_{i}\frac{dc_{i}}{dx}$$

Regarding single-layer membranes, at the interfaces for a specific gas species *i*, the following expressions should hold:

Feed side: *c*_1_ =* K_i_p*_1_; permeate side: *c*_2_ =* K_i_p*_2_

After integrating over the membrane thickness from 0 to *l*,

(17) $$J_{i}=\frac{D_{i}}{l}\left(c_{1}-c_{2}\right)=\frac{D_{i}K_{i}}{l}\left(p_{1}-p_{2}\right)$$

where *D_i_K_i_*/*l* is the gas permeance (thickness normalized permeability), and *D_i_*/*l* is the thickness normalized diffusivity coefficient.

Similarly, regarding dual-layer membranes, at the interfaces for a specific gas species *i*, the following expressions should hold:

Feed side: *c*_1_ =* K_i_*_1_*p*_1_; permeate side: *c*_2_ =* K_i_*_2_*p*_2_

After integrating over the membrane thickness from 0 to *l*,

(18) $$J_{i}=\frac{D_{i}}{l}\left(c_{1}-c_{2}\right)=\frac{D_{i}}{l}\left(K_{i1}p_{1}-K_{i2}p_{2}\right)$$

To obtain a similar expression with the single-layer membrane, the above equation can be changed as follows:

(19) $$J_{i}=\frac{D_{i}}{l}\left(c_{1}-c_{2}\right)=\frac{D_{i}}{l}\left(K_{i1}p_{1}-K_{i2}p_{2}\right)=\frac{D_{i}}{l}\frac{K_{i1}p_{1}-K_{i2}p_{2}}{p_{1}-p_{2}}\left(p_{1}-p_{2}\right)$$

where $\frac{K_{i1}p_{1}-K_{i2}p_{2}}{p_{1}-p_{2}}$ is considered the averaged solubility coefficient across the dual-layer membrane (*K_DLM_*). If *K_i_*_1_ = *K_i_*_2_, the transport equations for dual-layer membranes can be reduced for single-layer membranes. If *K_i_*_1_ >* K_i_*_2_, it is easily deduced that $\frac{K_{i1}p_{1}-K_{i2}p_{2}}{p_{1}-p_{2}}$ > *K_i_*_1_ > *K_i_*_2_. Conversely, however, if *K_i_*_1_ < *K_i_*_2_, we obtain $\frac{K_{i1}p_{1}-K_{i2}p_{2}}{p_{1}-p_{2}}$ < *K_i_*_1_ < *K_i_*_2_. This suggests that the configurations of dual-layer membranes tend to adjust the solubility gradient across the membrane.

Case (ii): hybrid model

In this scenario, we assume the following conditions: (i) both concentration and pressure in the membrane decrease gradually across the membrane thickness; (ii) the diffusion coefficients across each layer are comparable.

(20) $$J_{i}=-L_{i}\frac{d\mu_{i}}{dx}=-\frac{L_{i}RT}{c_{i}}\frac{dc_{i}}{dx}-\frac{L_{i}RT}{p_{i}}\frac{dp_{i}}{dx}$$

Similarly, we replace *L_i_RT*/*c_i_* by *D_i_* and replace *L_i_RT*/*p_i_* with Darcy’s law coefficient, *k_i_*.

(21) $$J_{i}=-D_{i}\frac{dc_{i}}{dx}-k_{i}\frac{dp_{i}}{dx}$$

In the case of a single-layer membrane, after integrating over the membrane thickness from 0 to *l*,

(22) $$J_{i}=\frac{D_{i}K_{i}}{l}\left(p_{1}-p_{2}\right)+\frac{K_{i}}{l}\left(p_{1}-p_{2}\right)=\left[\frac{D_{i}K_{i}}{l}+\frac{K_{i}}{l}\right]\left(p_{1}-p_{2}\right)$$

where membrane permeance (*P*) is the sum of the contributions from Fick’s law (*D_i_K_i_*/*l*) and Darcy’s law (*k_i_*/*l*).

Similarly, in the case of a dual-layer membrane, after integrating over the membrane thickness from 0 to *l*,

(23) $$J_{i}=\frac{D_{i}}{l}=\frac{D_{i}}{l}\left(K_{i1}p_{1}-K_{i2}p_{2}\right)+\frac{k_{i}}{l}\left(p_{1}-p_{2}\right)=\left[\frac{D_{i}}{l}\frac{K_{i1}p_{1}-K_{i2}p_{2}}{p_{1}-p_{2}}+\frac{k_{i}}{l}\right]\left(p_{1}-p_{2}\right)$$

Thus,

(24) $$P_{i}=\frac{D_{i}}{l}\frac{K_{i1}p_{1}-K_{i2}p_{2}}{p_{1}-p_{2}}+\frac{k_{i}}{l}$$

(25) $$K_{DLM}=\frac{K_{i1}p_{1}-K_{i2}p_{2}}{p_{1}-p_{2}}$$

As indicated previously, in the solution-diffusion model, if *K_i_*_1_ > *K_i_*_2_, we obtain *K_DLM_* > *K_i_*_1_ > *K_i_*_2_. Therefore, the configurations of the solubility gradient may play an important role in gas permeation properties for dual-layer or multi-layer membranes. It is worth noting that the dual-layer or multi-layer membrane in this study comprised a membrane with a layered structure and comparable diffusivities across each layer. The conclusions in this study may not be correct for asymmetric membranes with very different texture properties in each layer.

**Figure 7:**
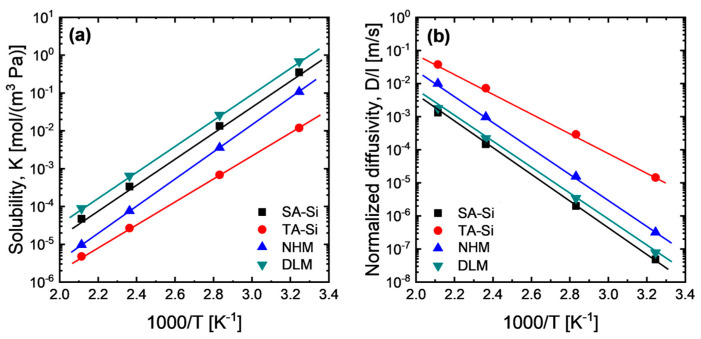
Temperature dependence of (**a**) solubility and (**b**) normalized diffusivity of CO_2_ for amine–silica membranes (*p*_1_ = 200 kPa, *p*_2_ = 100 kPa). The average solubility of DLM was calculated based on the hypothesized transport model for dual-layer membranes.

To verify the possible effect of the solubility gradient across each layer, we investigated CO_2_ solubility at a broad temperature range of the amine–silica membrane with a homogenous matrix (SA-Si, TA-Si, and NHM) and the average solubility of the DLM, as shown in Figure 7a. Apparently, the NHM showed CO_2_ solubility in between SA-Si and TA-Si due to the mixing effect. It is easier to speculate that the high-solubility material (SA-Si) defines the upper bound of the solubility of the hybrid material, while the low-solubility material (TA-Si) defines the lower bound. However, as calculated, the solubility of the dual-layer membrane can exceed the upper bound of the high-solubility material (SA-Si). This implies that the solubility gradient can be further adjusted based on the configurations of dual-layer or multi-layer membranes with different levels of solubility to the penetrated gas compared with these homogeneous membranes. Moreover, the high-solubility material should be configured as the topmost layer, while a moderate-solubility material should be a sublayer or transition layer to further enhance the solubility gradient. Indeed, generally, there is concentration (molar/volume) enrichment of the penetrated gas in both feed and permeate sides, particularly for high-condensable gases (e.g., CO_2_), based on a Boltzmann distribution:

(26) $$\frac{c_{a}}{c_{b}}=\exp\left(\frac{-\Delta H_{s}}{RT}\right)$$

Here, *c_a_* and *c_b_* are the adsorbed concentration and bulk (gas phase) concentration, respectively.

The concentration enrichment in the feed interface is mostly a positive effect for gas permeation, while the concentration enrichment in the permeate interface exerts a negative effect that may reduce the effective concentration gradient. Figure 7b summarizes the thickness-normalized diffusivity of the amine–silica membranes obtained after the disintegration of membrane permeance based on P = K*D/l. There are order differences in diffusivity between SA-Si and TA-Si membranes, which suggests that the DLM is not an ideal dual-layer membrane that can be well described by the dual-layer transport equations shown above. However, there is still room to discuss the contributions of solubility and diffusivity to the membrane permeance (or permeability), since the diffusivity coefficient is always an inconstant parameter linked with the relevant driving forces for flux. By comparing the normalized diffusivity among amine–silica membranes, one may find that the DLM with higher permeance than SA-Si and NHM showed even lower or comparable diffusivity, suggesting that the enlarged solubility gradient of DLM based on the configurations of dual-layer structure plays a more important role in CO_2_ permeation properties. It should be noted that, in this study, direct layer-specific sorption measurements on the actual dual-layer membranes were not conducted. Instead, gas adsorption isotherms of the corresponding xerogel powders (SA-Si and TA-Si) were employed to estimate the relative CO_2_ affinities of each component. While informative, these adsorption measurements on xerogels cannot resolve the spatial distribution of solubility within the layered membrane structure and thus cannot directly confirm the existence of a solubility gradient.

## 4. Conclusions

In this study, we successfully developed a hierarchical amine-functionalized silica membrane with engineered affinity gradients to address the intrinsic trade-off between CO_2_ adsorption capacity and diffusion kinetics in conventional homogeneous facilitated transport membranes. By strategically constructing a dual-layer architecture comprising a high-affinity top layer (SA-Si) and a low-affinity sublayer (TA-Si), we established a continuous solubility gradient that significantly enhances the driving force for CO_2_ permeation. Experimental gas permeation tests demonstrated that the DLM membranes achieved a CO_2_ permeance of 1.8 × 10^−7^ mol/(m^2^·s·Pa) at 35 °C, representing a 45% improvement over the homogeneous NHM, while maintaining exceptional CO_2_ selectivity. Theoretical modeling revealed that the solubility gradient amplified the effective Henry’s constants compared to SA-Si alone, with the interfacial chemical potential gradient reducing the apparent activation energy for CO_2_ diffusion. The hierarchical design decouples the conflicting requirements of strong CO_2_ binding and rapid transport. The combination of temperature-dependent permeation tests (35–200 °C) and dual-mode sorption modeling confirmed that the affinity gradient maintains stable operation under industrially relevant conditions. Our findings establish a universal framework for engineering affinity gradients in hybrid membranes, with potential applications extending to H_2_ purification and olefin/paraffin separations. Future work will focus on optimizing layer thickness ratios and expanding this architecture to metal–organic framework-based membranes, where the precise control of interfacial chemistry could further enhance separation factors. This study provides both fundamental insights into gradient-driven transport mechanisms and practical guidelines for scalable membrane fabrication in carbon capture systems. Although all permeation tests were conducted under dry conditions to isolate intrinsic transport properties, we recognize that humidity is inevitable in practical CO_2_ capture scenarios. The presence of water vapor is expected to enhance CO_2_ uptake in the amine-rich top layer via bicarbonate formation, potentially improving the overall separation performance. Future work will systematically evaluate the influence of humidity on transport behavior and stability of the DLM architecture under realistic flue gas conditions.

## Figures and Tables

**Table 1:** Activation energies of gas permeation and CO_2_/N_2_ selectivity at different temperatures for the amine–silica membranes prepared in this work.

Membranes	*E_p_* [kJ/mol]					CO_2_/N_2_ Selectivity [−]			
	He	H_2_	CO_2_	N_2_	CH_4_	35 °C	80 °C	150 °C	200 °C
SA-Si	12.8	12.6	9.8	14.1	14.9	10.7	10.7	7.2	6.3
TA-Si	7.4	7.4	0.2	10.1	9.5	20.8	14.6	7.9	5.5
NHM	12.6	12.2	7.3	15.0	14.6	14.0	12.3	7.5	4.9
DLM	12.7	12.0	8.3	13.2	11.8	10.7	9.6	7.4	5.3

## Data Availability

The data that support the findings of this study are available from the corresponding author upon reasonable request.
